# Induced Pluripotent Stem Cells and Periodontal Regeneration

**DOI:** 10.1007/s40496-015-0065-8

**Published:** 2015-09-16

**Authors:** Mi Du, Xuejing Duan, Pishan Yang

**Affiliations:** Shandong provincial key laboratory of oral tissue regeneration, Department of Periodontology, School of Stomatology, Shandong University, No.44-1 West Wenhua Rd., Jinan, 250012 People’s Republic of China; Department of Stomatology, Shandong Provincial Hospital Affiliated to Shandong University, No.324 Jingwu Rd., Jinan, 250000 People’s Republic of China

**Keywords:** Induced pluripotent stem cells, Periodontal regeneration, Tissue engineering

## Abstract

Periodontitis is a chronic inflammatory disease which leads to destruction of both the soft and hard tissues of the periodontium. Tissue engineering is a therapeutic approach in regenerative medicine that aims to induce new functional tissue regeneration via the synergistic combination of cells, biomaterials, and/or growth factors. Advances in our understanding of the biology of stem cells, including embryonic stem cells and mesenchymal stem cells, have provided opportunities for periodontal tissue engineering. However, there remain a number of limitations affecting their therapeutic efficiency. Due to the considerable proliferation and differentiation capacities, recently described induced pluripotent stem cells (iPSCs) provide a new way for cell-based therapies for periodontal regeneration. This review outlines the latest status of periodontal tissue engineering and highlights the potential use of iPSCs in periodontal tissue regeneration.

## Introduction

Periodontitis comprises a set of chronic inflammatory diseases affecting the periodontal supportive tissues (gingiva, alveolar bone, periodontal ligament, and cementum) and, if left untreatred, results in progressive loss of the alveolar bone around the teeth, resulting in loosening and subsequent loss of teeth. Conventional therapy of periodontitis concentrates on reduction of the bacterial load by mechanical and antimicrobial treatment, including scaling and root planing (SRP) or open flap debridement (OFD). These therapeutic procedures can effectively control inflammation and stop disease progression. Moreover, the application of surgical procedures such as guided tissue regeneration (GTR) and/or bioactive materials or molecules has attained some success to regenerate lost periodontal tissues [1••]. However, current regenerative procedures still have limitations in attaining complete and functional tissue regeneration, especially in advanced periodontal defects [2]. Tissue engineering, in which there are three major constituents, (1) biomaterials, (2) stem cells, (3) tissue growth factors, provides a new and better prospective for periodontal tissue regeneration [3]. Embryonic stem cells (ESCs) and mesenchymal stem cells (MSCs), including periodontal ligament stem cells (PDLSCs), bone marrow-derived MSCs (BMSCs), and adipose-derived stem cells (ADSCs) [4], are the most common cells used in periodontal tissue engineering. However, recently developed induced pluripotent stem cells (iPSCs) are increasingly attracting wide interest [5]. The present review will outline the latest status of periodontal tissue engineering and highlight iPSCs in terms of their properties and applications as well as the challenges and future prospects.

## Biomaterials

Biomaterials as scaffolds play pivotal roles in periodontal tissue engineering by serving as matrices for cell growth, proliferation, differentiation, and new tissue formation in three dimensions, and as carriers to convey cells and various tissue-inducing substances such as growth factors which are essential for tissue development. Various conventional materials have been investigated as scaffolds in periodontal tissue engineering. They fall into two broad classes: natural materials and synthetic materials. The available natural materials include collagen, gelatin, and modified polyscacchrides (e.g., chitosan). The synthetic materials include calcium phosphate (e.g., hydroxyapatite, tricalcium phosphate), bioactive glass, and some synthetic polymers [e.g., poly(glycolic acid)]. These materials show the characteristics of biocompatibility, controllable degradation, and tunable mechanical properties, but fall short in bioactivity. Recent developments indicate that the application of nanobiomaterials in periodontal tissue engineering improve the mechanical properties and incorporate the nanotopographic features that mimic the natural nanostructure of bone, and show higher bioactivity.

The concept of nanotechnology, raised by the quantum theorist and Nobel laureate Richard Feynman in 1959, is the manipulation of materials at the nanometer scale to fabricate “nanomaterials,” which have “internal or surface structures in one or more dimensions in the size range 1 ∼ 100 nm” [6]. Materials at this scale are endowed with a higher surface to volume ratio, and thereby have superior mechanical, magnetic, optical, and chemical properties to those of the other materials [7]. Their higher surface-to-volume ratio assists in effective adsorbtion of proteins which are beneficial to cell adhesion [8]. Moreover, the geometry of nano-scaffolds, the crystallinity and orientation of the polymer, can affect the affinities of proteins for such materials [9]. Surface roughness is another key characteristic affecting cell response. Obviously, nano-scaffolds possess different scales of surface roughness from conventional scaffolds. The studies demonstrated that cells might be more sensitive to changes in the surface roughness in the nanometer (<100 nm) compared with conventional micro- or macro- (>100 nm) regimes [10]. All these properties reflect superiority of nanostructured scaffolds in bone/periodontal tissue engineering comparing with conventional materials.

## Tissue Growth and Chemotactic Factors

The rationale for the use of growth factors for periodontal regeneration relies on the ability of these factors to enhance the proliferation and differentiation of PDLSCs and the other MSCs whether transplanted with tissue engineering construct or derived from host body. Recombinant growth factors can also enhance bone regeneration. For example, fibroblast growth factors (FGF), platelet-derived growth factors (PDGF), insulin-like growth factors (IGF), and bone morphogenetic proteins (BMP) have been used in clinical and pre-clinical trials for the treatment of large periodontal or infra-bony defects [11]. The topical application of enamel matrix derivative (which may contain a mixture of growth factors and other agents able to induce tissue repair and regeneration) with or without use of GTR can partially promote the formation of new periodontal tissues [12–14]. Considering the quick break down and short half-life period of tissue growth factors, controlled release and genetic approaches may overcome these pitfalls [15].

The principle of using chemotactic factors in periodontal regeneration relies on recruiting MSCs into periodontal defects [16]. These MSCs are not only derived from periodontitis involved local area but also from systemic circulation [17]. This technique, known as endogenous cell homing, would be less costly and complex than approaches that require substantial ex vivo cell manipulation and that use artificial vehicles for cell delivery, thereby having greater potential to provide new therapeutic options for in situ tissue regeneration [18].

The application of CXCL12 plays an important role in the recruitment of MSCs [19]. In addition, BMPs, basic fibroblast growth factor (bFGF), and PDGF-BB have been also demonstrated to have chemotactic effect on MSCs, of which bFGF possesses wider bioactivities than CXCL12. Schmidt et al. found that the chemotactic effect of bFGF on MSCs was stronger than CXCL12 [20]. Tasso et al. showed that the presence of bFGF in the culture medium during mouse MSCs expansion in vitro is a key factor for the selection of subpopulations inducing host regenerative responses [21]. Moreover, bFGF is related to maintenance of stem cell “stemness,” while preserving its differentiation potential [22]. Additionally, bFGF can exert strong proliferation enhancement on MSCs and osteoblasts [23]. All these activities of bFGF suggest that it may be an optimal choice for in situ periodontal tissue regeneration.

## Stem Cells

Stem cells are the foundation cells for every organ and tissue in the body, including the periodontium [24]. Three categories of stem cells, ESCs, MSCs, and the recently developed induced pluripotent stem cells, have been experimentally applied in enhancing bone/periodontal regeneration.

Embryonic stem cells are pluripotent cells which were derived from the inner cell mass of blastocyst-stage embryos. Pluripotency is the potential of one type of cell to differentiate into various kinds of cells, such as muscle cells, neural cells, and even germ cells [25]. Based on this property, ESCs can generate any type of cell to meet the requirements of different applications. ESCs are also capable of self-renewal; they can be semi-permanently cultured on feeder cells, which supply the necessary growth factors for ESCs. The experiments have shown that ESCs possess potential to differentiate towards fibroblastic and osteoblastic lineages in vitro [25, 26] and enhance the regeneration of periodontal furcation defects in a porcine model [27]. However, the number of approved human embryonic stem cell lines for research is limited and this has impacted on the development of these cells for treating human diseases [28]. Indeed, the use of human embryos is controversial, and the ethical considerations, risk of teratoma formation, and immunologic rejection following transplantation in patients all remain significant concerns [29].

MSCs derived from multiple tissue sources have been investigated in preclinical animal studies for periodontal regeneration therapy. The potential of BMSCs, ADSCs, dental pulp stem cells (DPSCs), stem cells from exfoliated deciduous teeth (SCED), PDLSCs, stem cells from apical papilla (SCAP), and progenitor cell populations from the dental follicle and gingiva for periodontal regeneration in a variety of animal models have been comprehensively reviewed by Han et al. [1••]. Furthermore, the application of MSCs in bone tissue engineering has moved to the preclinical stage, and an ex vivo cell manufacturing procedure for obtaining high quality, bioactive MSCs from human bone marrow has been approved by the US Food and Drug Administration (FDA) [30]. Nevertheless, the major obstacles that impede the use of MSCs in clinical practice lie in the heterogeneity of the isolated cell population and the inability to set up optimal growth and differentiation conditions to obtain and maintain required quality and quantity of cells. MSCs have a limited proliferation capacity, reduced the differentiation potential, and decreased protective factors during prolonged ex vivo subculturing and passages [31, 32]. Passage of cells, donor age, and aging-related disorders also significantly impair the survival and differentiation potential of MSCs [33–35]. One study has shown a significant age-related decrease in the growth rate of human-BMSCs from donors older than 50 years old as compared with younger donors [36]. In addition, their ability of self-renewal, osteogenic differentiation, and proliferation decreases due to diseases like osteoporosis and arthritis [37, 38]. There also are increasing concerns that exogenously infused MSCs can home to tumor microenviroment and exert promotive effect on tumor growth [39–41]. Thus, better understanding of the regulation mechanisms of self-renewal and “stemness” retaining is needed in order to sufficiently regulate MSC growth in vitro to produce necessary cell numbers and quality. The interactions between stem cells and the immune system or stem cells and osteoblast-osteoclast balance as well as controlling strategy of MSC malignant transformation in vivo should be further explored [1••].

## iPSCs

### Generation of iPSCs

Based on the hypothesis that the genes that have important significance in maintaining ESC identity also exert key effects in inducting pluripotency of somatic cells, Takahashi and Yamanaka [42] introduced different combinations of selected 24 genes, which were important transcripts of ESCs and oncogenes, as candidate reprogramming factors into mouse embryonic fibroblasts in order to screen proper reprogramming factors via the Fbx15-Neo reporter system. They found that after introduction of the retroviral mediated factors *Oct3/4*, *Sox2*, *Klf4*, and *c-Myc*, mouse embryonic fibroblasts were reprogrammed into ES cell-like cells called iPSCs. The generation of iPSCs from adult human dermal fibroblasts was also demonstrated by transfection of the same four factors [43]. Human iPSCs share similar biological characteristics with human ESCs including morphology, proliferation, surface antigens, gene expression, epigenetic status of pluripotent cell-specific genes, and telomerase activity. Moreover, these cells could differentiate to cell types of the three germ layers in vitro and in teratomas [44].

### Cell Sources for Deriving iPSCs and Approaches for Reprogramming

iPSCs have been derived from many different species, such as humans, mice, pigs, rabbits, rats, marmosets, and rhesus monkeys. The successfully reprogrammed cell types contain fibroblasts, marrow mesenchymal cells, gastric/intestinal epithelial cells, keratinocytes, hepatocytes, stomach cells, neural stem cells, pancreatic cells, blood/liver/neural progenitor cells, cord/peripheral blood cells, adipose stem cells, B and T lymphocytes, and so on [45–48], of which fibroblasts are the most commonly used parental somatic cell type for the generation of iPSCs. Between 2010 and 2012, the first reports were published on the production of iPS cell lines from human gingival fibroblasts and periodontal ligament fibroblasts by reprogramming using a retroviral transduction cocktail of OCT3/4, SOX2, KLF4, and c-MYC [49–51]. These induced cell lines expressed the human-ES (hES) cell-associated cell-surface antigens like SSEA3, SSEA4, GCTM-2, TG30 (CD9), and Tra-1-60, as well as the hESC marker genes OCT4, NANOG, and GDF3 [52]. Interestingly, in 2012, another research group established iPSCs from human periodontal ligament fibroblasts by introducing the human-ESC (hESC) markers OCT3/4, SOX2, NANOG, KLF4, and Lin28 through retrovirus transduction, even without the oncogene c-MYC [51], which was found to be responsible for tumors in iPSC chimeric mice [53]. In 2015, Umezaki et al. [54] demonstrated that human gingival integration-free iPSCs could be generated using episomal plasmid vectors without retroviral transduction. Compared with skin, gingival tissue is obtained more easily and gingival wound after sampling heals more quickly. Furthermore, reprogramming efficiency of mouse gingival fibroblasts is higher than that of mouse dermal fibroblasts [55]. These discoveries suggest that iPSCs derived from GFs represent an optimal and more practical cell-based tissue-regenerative treatment for periodontal diseases.

Since the first establishment of iPS cell line by Yamanaka in 2006, many scientists have made efforts to improve the efficiency and safety of the reprogramming process. Generally, the approaches for factor reprogramming include transgene and chemical reprogramming while methods for transgene reprogramming can be classified into three groups: RNA-based, DNA-based, and protein transduction (direct cell transduction). RNA-based reprogramming can be achieved by transfection of synthetic RNA, modified RNA, and micro RNA [56••]. DNA-based ways are most widely used, which include the use of viruses and plasmids. The very first way for cell reprogramming was by retroviral delivery of four transcription factors (*Oct4*, *Sox2*, *Klf4*, and *Myc*). In 2009, some researchers found direct protein transduction can improve inducing efficiency, but it is easily affected by the quality of recombinant proteins [57–59]. As for chemical cell reprogramming, the use of small-molecule compounds has been developed [51], and several reviews on small molecule drugs used for improving the generation of iPSCs are available [60, 61]. A variety of reprogramming methods to derive iPSCs and their advantages and disadvantages are shown in Table 1. Among available reprogramming methods, the easiest and most efficient method by now is the integration of reprogramming factors into the genome by retroviral or lentiviral transduction [43, 75].

**Table 1:** Advantages and disadvantages reprogramming methods to derive iPSCs

Approach			Cell type	Advantage	Disadvantage	Reference
	Forms	Vectors	Fibroblasts, neural stem cells, stomach cells, liver cells, keratinocytes, amniotic cells, blood cells, adipose cells, melanocytes, human T cells, β cells	No genomic integration, no premature silencing, inexpensive	Low efficiency, sequence-sensitive RNA replicase, difficulty in purging cells of replicating virus Genomic recombination, insertional mutagenesis	[62–65]
Transgene (OSKM)	DNA-based	Viruses (lentivirus, adenovirus, Sendai virus )				
		Transposon	Fibroblasts, mouse ESCs	Host-factor independent, wide chromosomal distribution, high efficiency	Gene mutations, genomic rearrangements	[66–68]
		Minicircle DNA	Human ASCs	High expression in mammalian cells, high transfection efficiency, stable ectopic transgene expression	Low expressions for transcription factors	[69]
	RNA-based	Micro RNA	Mouse fibroblasts, Human fibroblasts	Nonviral, nontranscription-factor	Multiple transfection, low efficiency	[70, 71]
		Modified RNAs	Human fibroblasts	Avoid the endogenous antiviral cell defense, very high efficiency	Technically complex	[65]
		Synthetic RNAs	Murine EFs, human epidermal keratinocytes	High efficiency	High and dose-dependent cytotoxicity	[65, 72]
	Recombinant Proteins		Human fibroblasts, mouse fibroblasts	Nongenome integration, easily controlled	Very low efficiency, unstable, easily affected by quality of proteins	[57–59]
Chemical approaches (small molecule compounds)			Mouse EFs	Nonimmunogenic, cost-effective, easy to handle, structural versatility, faster, more efficient, self-renewal promotion, controllable microenvironment	Time and dosage of specific biochemicals need to be optimized	[44, 61, 73, 74]

OSKM and similar factor names represent combinations of reprogramming factors: O, OCT4; S, SOX2; K, KLF4; M, c-MYC

*ESCs* embryonic stem cells, *EFs* embryonic fibroblasts

### Advantages of iPSCs Over Other Stem Cells

The serious ethical concerns associated with ESC application, and limited proliferation and differentiation potential of MSCs, has prompted efforts to genetically reprogram somatic cells to generate iPSCs. On the first hand, these cells have similar biological features to ESCs without any ethical concerns associated with ESCs [43, 76–78]. Although ESCs and iPSCs both carry tumorigenic properties, raising a significant safety challenge in the use of these cells for regenerative therapies, the most important advantage of iPSCs compared to ESCs is the possibility to use mature somatic cells from patients who suffer from defined diseases genetically [79–81]. In addition, human-iPSCs can differentiate into all cell types of three germ layers in vitro and in vivo [43]. Overall, iPSCs overcome major ethical concerns about hESCs such as the destruction of human embryos and oocytes. In particular, the clinical use of iPSCs is expected to solve the problems of immune rejection. On the other hand, iPSCs have a greater proliferation capacity than traditional MSCs so that they can expand into large numbers before in vitro differentiation and transplantation while remaining similar or superior multipotent differentiation potential to their parental MSCs [82, 83].

### iPSCs Used in Periodontal Regeneration

**Fig. 1:**
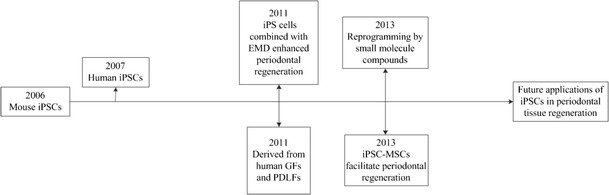
History of development for iPSCs in the field of periodontal regeneration

iPSCs now provide a novel approach to the field of tissue engineering. Recently, some studies have shown that iPSCs are a promising source of stem cells to be used for periodontal tissue regeneration therapy. In general, the rationale of applying iPSCs in periodontal regeneration includes the following aspects: (1) iPSCs can be induced from dental derived cells, such as gingival fibroblasts and periodontal ligament fibroblasts; (2) iPSCs can differentiate to osteogenic cells after stimulated by certain factors; (3) combining with or without the scaffolds, iPSCs can facilitate the healing of man-made periodontal bone defect and form new periodontal tissues like alveolar bone, cementum, and periodontal ligament. The development of iPSCs in the field of periodontal regeneration was summarized in Fig. 1.

#### iPSC Differentiation Toward Osteoblasts

In 2012, it was reported the murine iPSC-derived cells can differentiate to osteoblasts with the help of TGF-beta family, bFGF or BMP-2 [84]. Recent studies have reported that functional MSCs which were derived from human iPSCs could express characteristic MSC markers, differentiate into osteoblasts, adipocytes, and chondrocytes, and promote vascular and muscle regeneration [32]. Our group previously found that when iPSCs were stimulated with enamel matrix-derived (EMD) gel, the mRNA expression level of Runt-related transcription factor 2 (Runx2), a key transcription factor expressed during osteogenic differentiation, greatly increased in EMD-stimulated media; thus, EMD gel can promote the differentiation of iPSCs to osteogenic cells [85•]. Similarly, another study showed that iPSC-MSCs had good ability for attachment, proliferation, and osteogenic differentiation when attached on calcium phosphate cement (CPC) scaffold, showing high gene expressions of osteogenic markers including osteocalcin, alkaline phosphatase (ALP), collagen type I, and Runx2; hence, the iPSC-MSC-CPC construct is predicted promising to promote bone regeneration in periodontal or craniofacial repairs [86]. Overall, iPSCs have the potential to differentiate into osteogenic cells.

### iPSCs Promote Periodontal Regeneration

The development of new cementum with periodontal ligament fibers connected to alveolar bone is the main goal of periodontal regeneration [86, 87]. Ideally, regenerated periodontal ligament fibers are inserted into the newly formed cementum to connect the root surface and alveolar bone. Since iPSCs can be used as seed cells in tissue engineering, some researchers have revealed that mouse iPSCs combined with scaffolds such as EMD gel can promote the formation of alveolar bone, cementum, and periodontal ligament, thus enhancing the repair and healing of mouse periodontal defects [85•]. Another study has demonstrated that iPSCs can be induced to differentiate into MSC-like cells [88•]. These cells fulfill the International Society of Cellular Therapy’s minimal criteria for defining multipotent MSCs: they had plastic adherent properties, expressed key MSC-associated markers, and had the capacity to undergo tri-lineage differentiation. Furthermore, the generated human iPSC-MSC-like cells had the capacity to facilitate periodontal regeneration in a rat periodontal defect model, including newly formed fibrous tissue, newly formed mineralized tissue, and newly formed PDL-like tissues [88•]. Similarly, Yang et al. demonstrated that rat iPSCs could be induced to differentiate to MSCs, and intravenous and topical administration of these cells that had been transfected with tumor necrosis factor alpha-stimulated gene-6 (TSG-6), which has strong anti-inflammatory effect, was capable to decrease inflammation and inhibit alveolar bone resorption in rat experimental periodontitis [89].

### The Challenges and Future Perspectives Regarding iPSCs

At present, many limitations still affect the possible applications of iPSCs in clinical medicine. The main hindrances are related to the reprogramming efficiency, biological safety, and large-scale expansion and directed differentiations.

No matter what the reprogramming methods are, one of the main obstacles in reprogramming iPSCs is the inherent low efficiency of complete reprogramming [90]. Generally speaking, there are gene expression differences between human-iPSCs (hiPSCs) and hESCs or among different iPSC lines. One report has shown that gene expression of the donor cell type significantly contributes to the differences among hiPSCs and hESCs. Specifically, further analysis reveals that gene expression of fetal fibroblast-derived hiPSCs is closer to that of hESCs, followed by adipose, neonatal fibroblast, and keratinocyte-derived hiPSCs [91]. Therefore, the optimal choice of original cell type for iPSC reprogramming and the ideal protocols for high efficient complete reprogramming are the prerequisites for future clinical transformational study.

Biological safety is another main concern in relation to the application of iPSCs in tissue regeneration, including periodontal regeneration. First, the integration of reprogramming factors into the genome by retroviral or lentiviral transduction is the easiest and most efficient method by now for reprogramming iPSCs. However, the use of cells containing viruses brings up the possibility that viruses integrate into host chromosomes and lead to replication-induced DNA mutation [92] and potentially malignant transformations [93]. Additionally, the existence of viruses may stimulate immunological reaction [94]. Second, the principles of iPSCs in regenerative medicine rely on their ability to self-renew and to differentiate to cells of the three germ layers. However, it is these properties that predispose iPS to be tumorigenic and therefore hinder the clinical applications of these cells [95]. These issues highlight the need for generating virus-free iPSCs that are functionally identical to true ESCs [96].

Another handicap for using iPSCs in regenerative medicine is the deficiency of large-scale expansion and directed differentiation approaches. Obtaining sufficient iPSCs and directing them to differentiate into osteoblasts, cementoblasts, and periodontal fibroblasts are prerequisites for successful periodontal regeneration, and although there have been some evidences showing the strong potentials of iPSCs in cell-based periodontal regenerative therapy [85•, 88•], the ability of iPSC differentiation to cementoblasts, most key cells for periodontal regeneration, remains to be explored.

Finally, whether or not iPSCs possess immunoregulatory properties, which is regarded as an important aspect of mechanisms of MSCs, has not been explored and challenge also remains to identify the best combination of iPSCs, biomaterials, and growth factors for various clinical situations.

## Conclusion

The significant ethical concerns accompanied with the clinical use ESC in humans and limited proliferation and differentiation abilities of MSCs suggest that iPSCs may be a good alternative cell source for use in regenerative medicine. Although studies on the use of iPSCs for periodontal regeneration are in their early stages, iPSCs-based therapy strategies will have solid background and good prospectives for clinical periodontal regenerative treatment. The main endeavors should be to strengthen the reprogramming efficiency, assure biological safety, and optimize the strategies of large-scale expansion and directed differentiations.
